# Incidence trend and conditional survival estimates of gastroenteropancreatic neuroendocrine tumors: A large population‐based study

**DOI:** 10.1002/cam4.1598

**Published:** 2018-06-05

**Authors:** Qing Zhong, Qi‐Yue Chen, Jian‐Wei Xie, Jia‐Bin Wang, Jian‐Xian Lin, Jun Lu, Long‐Long Cao, Mi Lin, Ru‐Hong Tu, Ze‐Ning Huang, Ju‐Li Lin, Ping Li, Chao‐Hui Zheng, Chang‐Ming Huang

**Affiliations:** ^1^ Department of Gastric Surgery Fujian Medical University Union Hospital Fuzhou China; ^2^ Department of General Surgery Fujian Medical University Union Hospital Fuzhou China; ^3^ Key Laboratory of Ministry of Education of Gastrointestinal Cancer Fujian Medical University Fuzhou China; ^4^ Fujian Key Laboratory of Tumor Microbiology Fujian Medical University Fuzhou China

**Keywords:** age, conditional survival, gastroenteropancreatic neuroendocrine tumors, incidence trends, SEER stage, Surveillance

## Abstract

Given the rarity and indolent clinical course of gastroenteropancreatic neuroendocrine tumors (GEP‐NETs), conditional survival might be the most suitable parameter for cancer survivors who wish to receive accurate prognostic information during follow‐up. We have explored the updated incidence trend and the conditional survival of patients with GEP‐NETs. Incidence trends from 2000 to 2014 were determined through an assessment of patients in the SEER cancer registry. Patients diagnosed between 1988 and 2011 were included in the conditional survival analysis, and the 3‐year conditional cancer‐specific survival (CCS3) was computed. The incidence of GEP‐NETs, which is far higher than the incidence of many malignant tumors, is still increasing steadily (annual percentage change = 4.4). The risk of death from NETs is dynamic over time, and most deaths occur in the first 3 years after diagnosis. Patients with gastric, rectal, or appendiceal NETs hardly exhibit any excess mortality (CCS3 > 95%) given that they have already survived until a defined time‐point within 10 years. The initial difference between each age group basically disappeared with an extension of the survival time since the initial diagnosis of gastric, appendiceal, or rectal NETs, but the difference persisted for tumors at other sites. Although patients with advanced‐stage or higher‐grade tumors have a worse survival at diagnosis than patients with early‐stage or lower‐grade tumors, the difference diminishes and might even disappear over time. For GEP‐NETs that are rare but exhibit slow growth, clinically relevant variations in conditional survival were observed based on the time since diagnosis. Therefore, conditional survival can serve as a guideline that can be used by cancer survivors to plan their future and doctors to plan surveillance schedules.

## 1 INTRODUCTION

Neuroendocrine tumors (NETs) comprise a heterogeneous group of rare tumors that arise from cells throughout the diffuse endocrine system. Gastroenteropancreatic NETs (GEP‐NETs), which are the main subtype of NETs,^1^ can secrete peptides and neuroamines that cause distinct clinical syndromes, including carcinoid syndromes.^2^, ^3^, ^4^

As demonstrated by the mounting number of articles published each year (over 4000 articles have been reported every year in the last decade),^5^ the worldwide concern about NETs has been increasing, which is probably due to the reported increase in its incidence.^6^, ^7^, ^8^ However, updated data that focus on the incidence trend of GEP‐NETs are lacking. Moreover, given the rarity and indolent clinical course of GEP‐NETs, the number of NETs survivors has increased. Nevertheless, only a few relevant statistics are available to inform patients who have survived for a certain period after they are diagnosed with NETs of their prognosis at any given time. Due to a lack of related evidence, the current consensus guidelines recommend that patients with NETs after treatment of the primary tumor undergo a CT or MRI scan every 6‐12 months to monitor disease progression, but no definite guidance regarding the optimal surveillance time has been established. The current guidelines stipulate that monitoring should be performed for 10 years after treatment.^3^, ^9^, ^10^, ^11^ Conventional survival curves after cancer diagnosis provide a rather grim outlook because most patients die within the first few years after diagnosis. Conditional survival (CS) is an important index in this respect,^12^ and more relevant information can be provided to cancer survivors for personal health‐related planning and to clinicians for continued cancer surveillance.^13^

To the best of our knowledge, no previous study has evaluated CS among patients with GEP‐NETs. Therefore, the aim of this study was to explore the updated incidence trend of GEP‐NETs and to assess the 3‐year conditional cancer‐specific survival (CCS3) among patients after diagnosis with GEP‐NETs using data from the surveillance, epidemiology, and end results (SEER) cancer registry.

## 2 PATIENTS AND METHODS

### 2.1 Patients

As a population‐based cancer registry that collects cancer incidence and survival data from 18 regional population‐based registries, the SEER database covers approximately 27.8% of the US population (based on the 2010 census). We screened the cases of GEP‐NETs using the newest databases “Incidence ‐ SEER 18 Regs Research Data + Hurricane Katrina Impacted Louisiana Cases, Nov 2016 Sub (2000‐2014) <Katrina/Rita Population Adjustment>” for incidence data and “Incidence ‐ SEER 18 Regs Research Data + Hurricane Katrina Impacted Louisiana Cases, Nov 2016 Sub (1973‐2014 varying)” for survival data. The Site and Morphology of Collaborative Stage Data Collection System (CS Schema v0204+) was used to identify GEP‐NET cases. The following codes were used for identification of the histological type and tumor location: NETStomach, NETSmallIntestine, NETAmpulla, CarcinoidAppendix, NETColon, and NETRectum. Due to the lack of specific codes that correspond to pancreatic NETs (P‐NETs), we used ICD‐O‐3 histology codes to identify patients with P‐NETs, as detailed in a prior publication.^14^

### 2.2 Incidence trend analysis

Patients with GEP‐NETs who were diagnosed between 2000 and 2014 were included in the incidence trend analysis. All incidence data were adjusted according to age and were standardized according to the 2000 US standard population. In addition, we used the weighted least squares method to calculate the annual percent change (APC) using the SEER*Stat software.^15^

### 2.3 Conditional survival (CS) analysis

We selected patients with GEP‐NETs that were diagnosed between 1988 and 2011. The study dates were chosen based on the availability of complete data after 1988, and 2011 was chosen as the last year of diagnosis to allow for at least 3 years of actual follow‐up. We included patients from the SEER database based on the following characteristics: microscopic confirmation of the tumor, presence of single primary tumor (one primary or the first of at least 2 primaries), availability of complete staging information, and survival for more than 1 month.

Conditional cancer‐specific survival (CCS), which originated from conditional probability in biostatistics, can be calculated using the Kaplan‐Meier method or the life table method. The estimate of CCS3 was defined as the probability of remaining free of cancer mortality for an additional 3 years given that a patient had survived for *x* years and was calculated as follows: *x* − CCS3 = CSS (*x* + 3)/CSS (*x*), where CSS is the cancer‐specific survival.^16^ Variances in conditional probabilities were estimated using the formula established by Skuladottir et al^17^ Additionally, 95% confidence intervals (CIs) were determined with the assumption that the CCS rates follow a normal distribution. The hazard curve for CSS was plotted using kernel density smoothing.^18^

Survival estimates were computed for 5 age groups (younger than 44 years, 45‐54 years, 55‐64 years, 65‐74 years, and older than 75 years), except for patients with appendiceal NETs (A‐NETs), who were subdivided into 2 age groups (younger than 44 years and older than 45 years). Tumor staging was recorded based on the SEER database using the expanded extent of disease coding.^19^ Localized disease was defined as tumors that were confined within an organ with no obstruction of or invasion into regional structures. Regional disease included spread into surrounding structures, blood vessels, or local lymph nodes. Distant disease included extension or metastases into other organs or lymph nodes. Notably, the SEER grading system classifying tumors into well differentiated (SEER grade I), moderately differentiated (SEER grade II), poorly differentiated (SEER grade III), and undifferentiated/anaplastic (SEER grade IV) relies on histologic differentiation, which is different from the 2010 WHO grading nomenclature. Therefore, Grade III and Grade IV were combined into 1 category, as previously reported.^4^, ^6^, ^20^

Cancer patients had no excess mortality when the CCS3 reached 100%, and no difference in survival was observed between cancer patients and the general population. Excess mortality was classified as follows: substantial, little, and hardly any based on CCS3 estimates of <90%, 90%‐95%, and >95%, respectively.^12^

All the statistical analyses were performed using SPSS software, version 18.0 for Windows (SPSS Inc., Chicago, IL, USA) and R software (version 3.4.0). All the tests were 2‐sided, with the significance level set to *P* < .05.

## 3 RESULTS

### 3.1 Incidence

**Figure 1:**
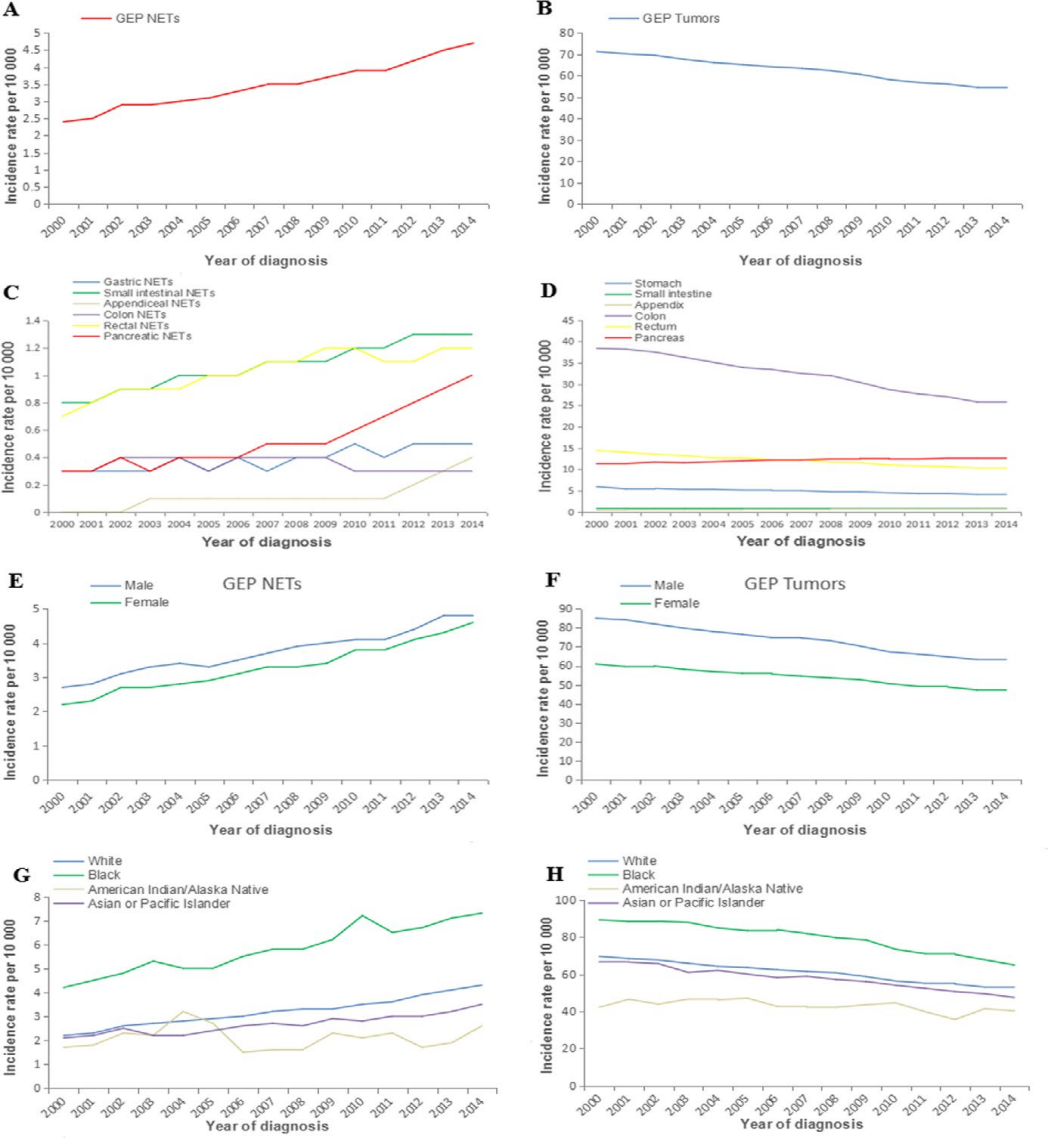
Annual age‐adjusted incidence rates of all GEP‐NETs (A) and all GEP neoplasms (B). Annual age‐adjusted incidence rates of GEP‐NETs (C) and GEP neoplasms (D) by site. Annual age‐adjusted incidence rates of GEP‐NETs (E) and GEP neoplasms (F) by gender. Annual age‐adjusted incidence rates of GEP‐NETs (G) and GEP neoplasms (H) by race (2000‐2014)

Between 2000 and 2014, 45 203 patients diagnosed with GEP‐NETs (based on a population size of 1 257 898 299) were identified by searching the SEER database. The year and age distribution of the patients are reported in Figure S1. The diagnostic age of most patients was between 50 and 64 years. The age‐adjusted incidence rates of GEP‐NETs significantly increased over time, with an APC of 4.5 [95% CI 4.2‐4.8], but the opposite was observed for the trends of all GEP malignancies (APC = −2.0, 95% CI −2.2 to −1.9; Figure 1A,B). Similarity could be observed across sex and race categories (Figure 1C‐F), except for GEP‐NETs in American Indian/Native Alaskan populations, which showed a stable incidence (APC = 0, 95% CI −2.9 to 3.0). With regard to GEP sites in patients diagnosed from 2000 to 2014, the growth was significant for A‐NETs (APC = 22.3, 95% CI 17.5‐27.2) and P‐NETs (APC = 9.8, 95% CI 8.5‐11.1; Figure 1G,I), but only NETs of the colon exhibited a constant incidence trend (APC = −0.5, 95% CI −1.6 to 0.5). These specific findings of GEP‐NETs are contrary to the trends in all malignant gastric tumors (APC = −2.4, 95% CI −2.6 to −2.2), colon malignancies (APC = −3.1 95% CI −3.3 to −2.8), and rectal cancers (APC = −2.4, 95% CI −2.5 to −2.2). Additionally, the findings are in accordance with the upward trend for all tumors of the small intestine (APC = 0.3, 95% CI 0.0‐0.6), appendix (APC = 3.4, 95% CI 2.8‐4.1), and pancreas (APC = 0.8, 95% CI 0.6‐1.0). Based on the SEER data, the incidence rates of GEP‐NETs and all gastroenteropancreatic cancers were determined to equal 3.5 and 62.1, respectively, per 100 000 per year over the same period (2000‐2014), which means that NETs represent 5.84% of all newly diagnosed GEP cancers.

### 3.2 Estimates of the 3‐year conditional cancer‐specific survival (CCS3)

**Figure 2:**
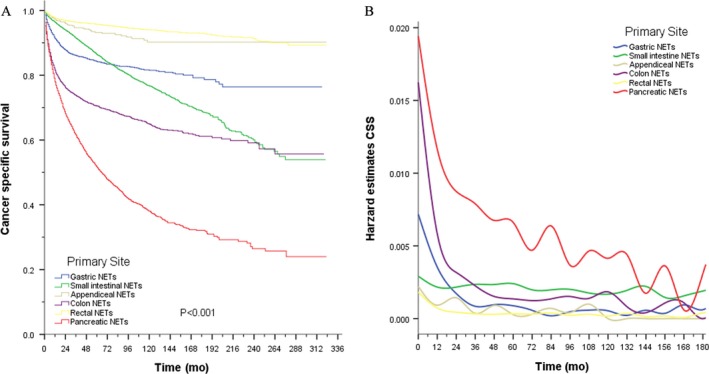
Kaplan‐Meier plots of GEP‐NETs (A) and hazard estimate of cancer‐specific death among the entire cohorts (B) according to site. CSS, cancer specific survival

Over the study period (1988‐2011), 28 056 patients met the inclusion criteria. The sociodemographic characteristics and clinicopathological features of the patients are presented in Table S1. Within this cohort, 2608 patients had gastric NETs (G‐NETs), 8637 patients had small intestinal NETs (SI‐NETs), 578 patients had A‐NETs, 2992 patients had colonic NETs (C‐NETs), 9290 patients had rectal NETs (R‐NETs), and 3951 patients had P‐NETs. The Kaplan‐Meier plots also demonstrated important survival differences based on different primary sites (*P* < .001). Additionally, the risk of NET‐specific death varies with time because most of the deaths occurred in the first 3 years after diagnosis for all sites (Figure 2). The actuarial CSS over the 3 years after diagnosis and CCS3 for those who had already survived 1‐10 years after diagnosis are presented at Figure S2. We evaluated the CSS patterns according to tumor site and age (Figure S3). Survival curves were then evaluated according to SEER stage and tumor grade, respectively (Figures S4 and S5).

**Figure 3:**
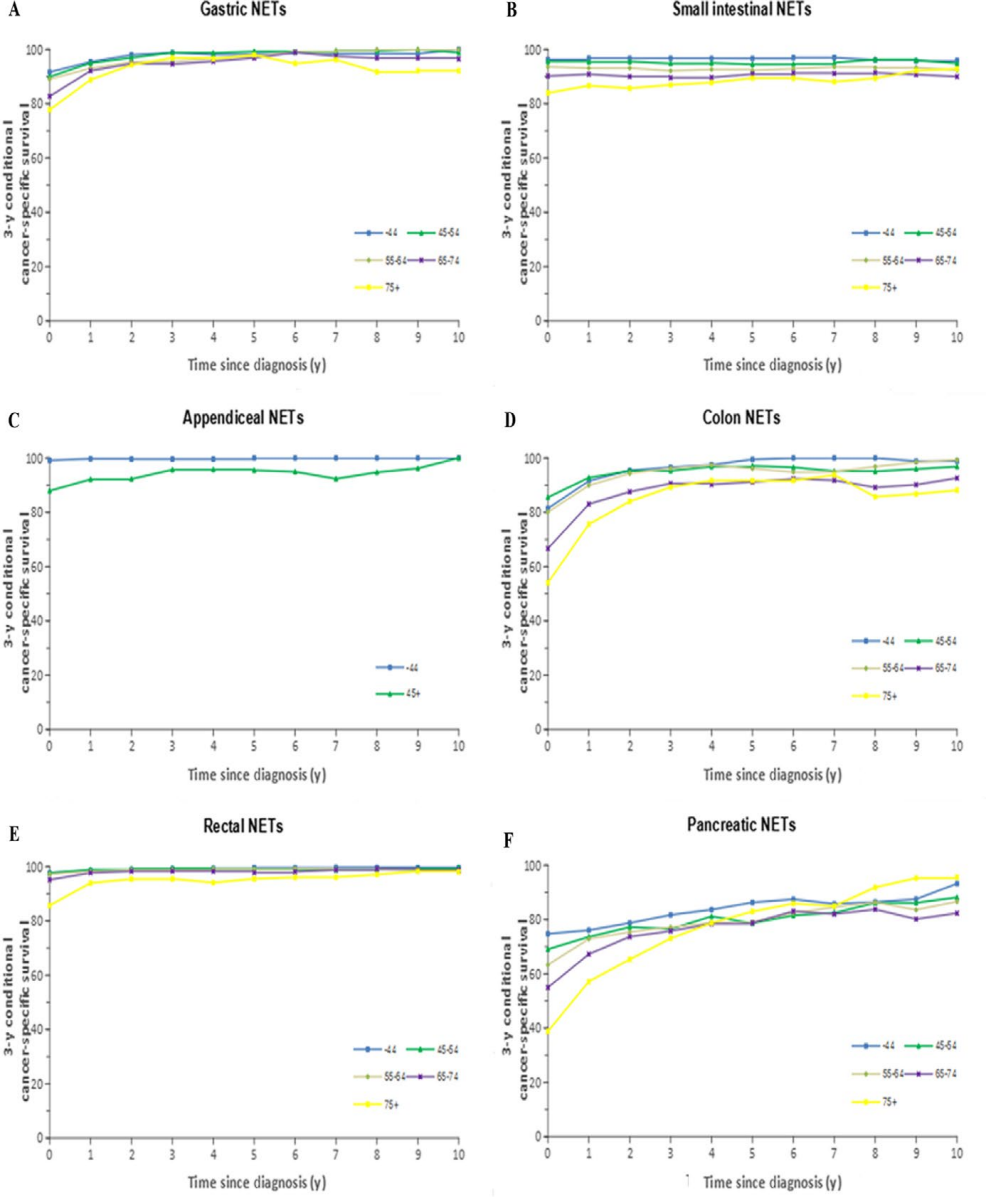
Three‐year cancer‐specific survival for every additional year survived after an initial diagnosis of cancer according to age groups: A, gastric NETs; B, small intestinal NETs; C, appendiceal NETs; D, colonic NETs; E, rectal NETs; and F, pancreatic NETs

**Figure 4:**
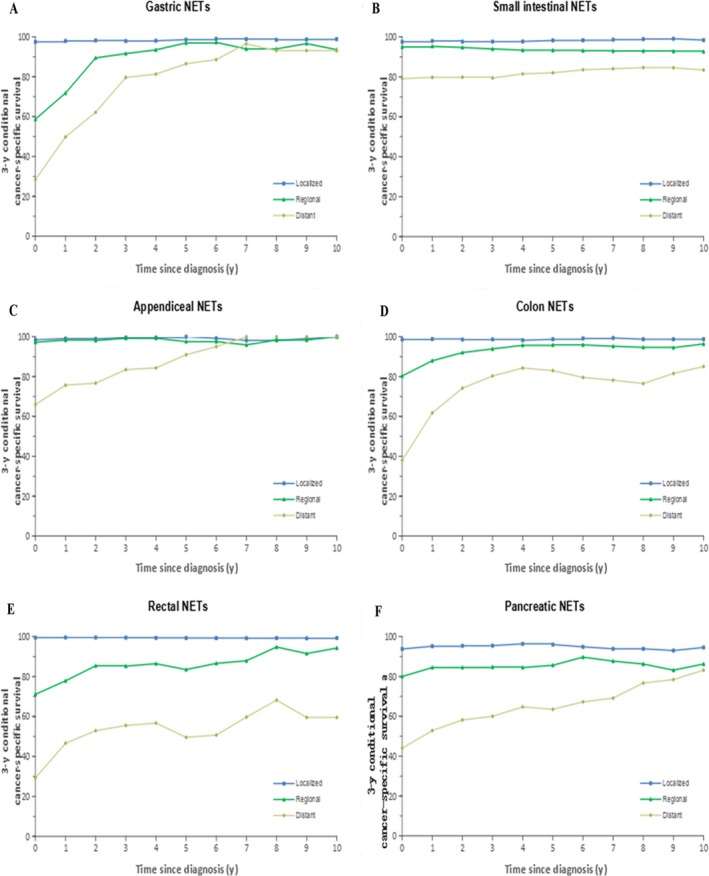
Three‐year cancer‐specific survival for every additional year survived after an initial diagnosis of cancer according to SEER stage groups: A, gastric NETs; B, small intestinal NETs; C, appendiceal NETs; D, colonic NETs; E, rectal NETs; and F, pancreatic NETs

**Figure 5:**
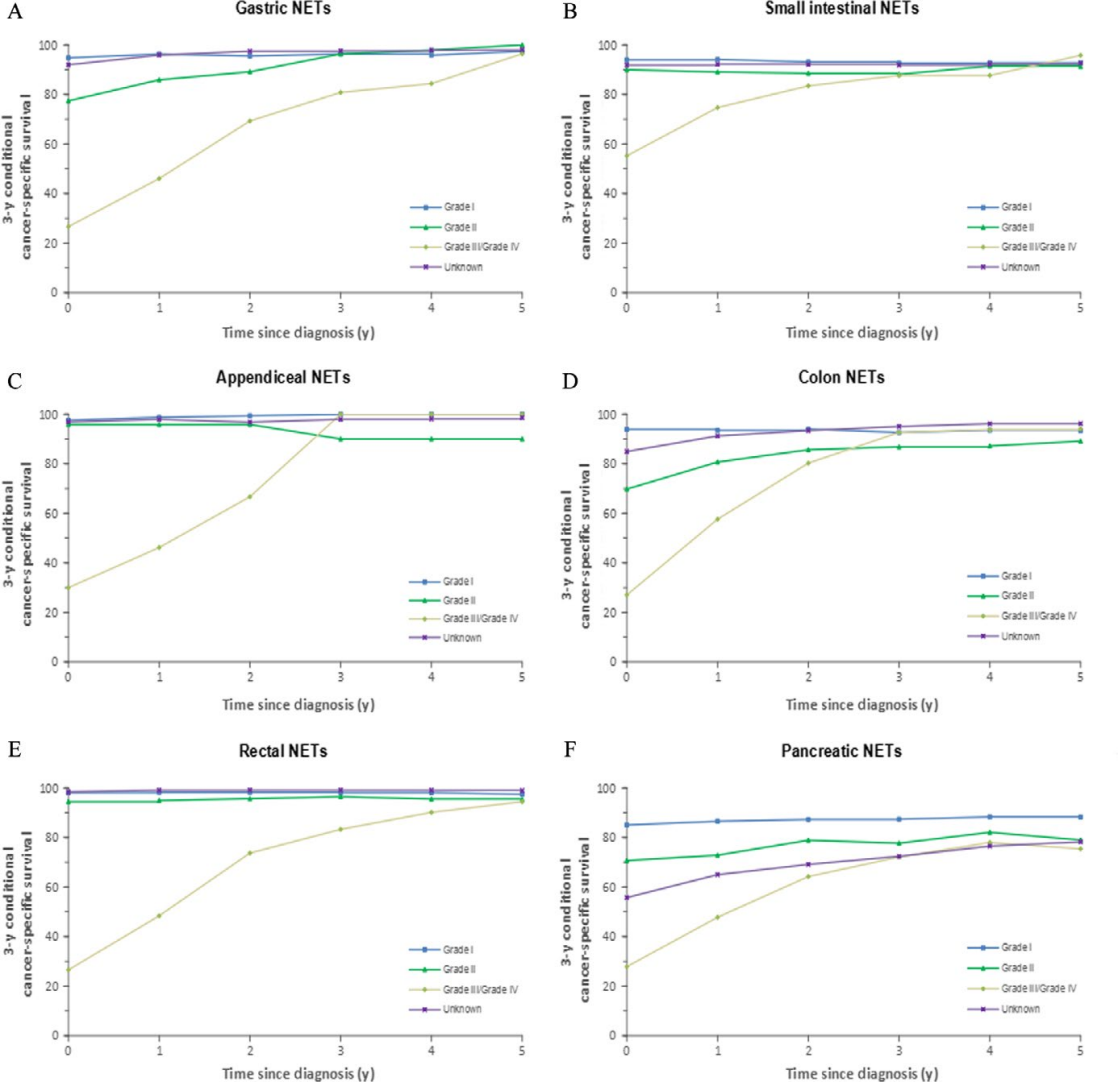
Three‐year cancer‐specific survival for every additional year survived after an initial diagnosis of cancer according to tumor grade groups: A, gastric NETs; B, small intestinal NETs, C, appendiceal NETs; D, colonic NETs; E, rectal NETs; and F, pancreatic NETs

Table 1 summarizes the number of patients included in the conditional period survival analysis after 5 and 10 years for each tumor site and age and the CS after 5 and 10 years. Additionally, the number of years after diagnosis when mortality showed little or hardly any change in a group of patients (when the CCS3 exceeds 90% or 95%, respectively) was determined. Table 1 shows the number of patients available for the conditional period survival analysis for each tumor site and age after they had survived for 5 and 10 years and for the CCS3 after 5 and 10 years. Moreover, the number of years after diagnosis when patients exhibit little or hardly any excess mortality (when CCS3 exceeds 90% or 95%, respectively) was demonstrated. Table 2 shows the relevant results by stage based on the SEER staging system. Figure 3 shows the CCS3 for each additional year of survival in patients with tumors at each site according to age, whereas Figures 4 and 5 show the results according to stage and grade, respectively.

**Table 1:** Number of patients available for conditional period survival analysis after 5 and 10 years for each site and age, and conditional survival after 5 and 10 years

Tumor Site	Age (years)	No. of patients available for conditional period survival analysis after year		3‐year conditional cancer‐specific survival (%)					
		5	10	At 5 years	95% CI	At 10 years	95% CI	>90% from year	>95% from year
Stomach	−44	234	114	98.5	96.8‐100.2	100.0	100‐100	0	1
	45‐54	316	134	99.3	98.3‐100.3	98.8	96.4‐101.2	1	2
	55‐64	333	129	97.5	95.6‐99.4	100.0	100‐100	1	2
	65‐74	274	91	96.9	94.5‐99.3	96.5	91.3‐101.6	1	4
	75+	187	48	98.0	95.6‐100.3	92.1	82.7‐101.5	2	3
Small Intestine	−44	591	322	96.7	95‐98.3	95.9	93.4‐98.4	0	0
	45‐54	1192	534	94.5	93‐96	94.9	92.6‐97.2	0	0
	55‐64	1505	603	92.3	90.6‐93.9	92.3	89.6‐95.1	0	—
	65‐74	1240	421	90.9	88.9‐92.9	90.0	86‐93.9	0	—
	75+	718	166	89.5	86.5‐92.4	92.8	87.5‐98.2	9	—
Appendix	−44	209	105	100.0	100‐100	100.0	100‐100	0	0
	45+	111	45	95.5	90.9‐100	100.0	100‐100	1	3
Colon	−44	192	110	99.5	98.4‐100.5	98.8	96.5‐101.1	1	2
	45‐54	448	210	97.1	95.4‐98.9	96.8	94‐99.7	1	2
	55‐64	462	203	96.1	94‐98.2	99.3	98‐100.6	2	3
	65‐74	308	127	91.2	87.5‐94.9	92.6	87.2‐98	3	—
	75+	170	57	91.6	86.4‐96.8	88.1	78‐98.2	4	—
Rectum	−44	1095	630	99.6	99.1‐100	99.6	99.1‐100.1	0	0
	45‐54	2315	1026	99.0	98.6‐99.5	99.6	99.2‐100.1	0	0
	55‐64	1875	825	99.0	98.5‐99.5	98.7	97.9‐99.6	0	0
	65‐74	1011	479	97.8	96.7‐98.8	98.6	97.4‐99.9	0	0
	75+	291	98	95.5	92.8‐98.2	98.0	94.1‐101.9	1	2
Pancreas	−44	325	125	86.3	81.5‐91.1	93.2	87.9‐98.5	10	—
	45‐54	397	124	78.6	72.9‐84.4	88.1	80.9‐95.4	—	—
	55‐64	358	98	79.1	72.9‐85.2	86.6	78.1‐95.2	—	—
	65‐74	227	63	78.7	71.1‐86.4	82.4	69.4‐95.3	—	—
	75+	81	18	82.9	70.9‐95	95.6	75.5‐115.7	8	9

Included in the analysis is the number of years after diagnosis when a group of patients appear to exhibit little excess mortality (when 3‐year conditional survival exceeds 90%) or hardly any excess mortality (when 3‐year conditional survival exceeds 95%). Conditional survival rate of >90% or >95% not reached within available follow‐up period with reliable estimates for conditional survival.

**Table 2:** Number of patients available for conditional period survival analysis after 5 and 10 years for each site and stage, and conditional survival after 5 and 10 years

Tumor site	Stage	No. of patients available for conditional period survival analysis after year		3‐year conditional cancer‐specific survival (%)					
		5	10	At 5 years	95% CI	At 10 years	95% CI	>90% from year	>95% from year
Stomach	Localized	1225	476	98.6	97.8‐99.3	98.9	97.7‐100	0	0
	Regional	73	25	96.9	92.6‐101.3	93.5	80.5‐106.6	3	5
	Distant	46	15	86.5	73.7‐99.3	93.1	79.1‐107.1	7	7
Small Intestine	Localized	2012	840	98.2	97.6‐98.9	98.4	97.3‐99.5	0	0
	Regional	2085	815	93.3	92‐94.6	92.6	90.3‐95	0	1
	Distant	1149	391	82.0	79‐85	83.4	78.2‐88.6	—	—
Appendix	Localized	187	85	100.0	100‐100	100.0	100‐100	0	0
	Regional	109	54	97.5	94.1‐101	100.0	100‐100	0	0
	Distant	24	11	91.0	77.8‐104.1	100.0	100‐100	5	6
Colon	Localized	818	394	98.7	97.8‐99.6	98.7	97.5‐100	0	0
	Regional	523	234	95.9	93.9‐97.8	96.4	93.6‐99.1	2	4
	Distant	239	79	83.0	76.6‐89.3	85.0	74.8‐95.3	—	—
Rectum	Localized	6453	3022	99.3	99.1‐99.5	99.3	99‐99.7	0	0
	Regional	91	29	83.5	73.1‐93.9	94.3	82.8‐105.8	8	—
	Distant	43	7	49.6	31.4‐67.7	59.5	21.1‐97.9	—	—
Pancreas	Localized	464	163	96.0	93.8‐98.2	94.5	90.3‐98.7	0	1
	Regional	425	149	85.6	81.3‐89.9	86.2	79.1‐93.4	—	—
	Distant	499	116	63.5	56‐71	83.1	73.9‐92.3	—	—

Included in the analysis is the number of years after diagnosis when a group of patients appear to exhibit little excess mortality (when 3‐year conditional survival exceeds 90%) or hardly any excess mortality (when 3‐year conditional survival exceeds 95%). Conditional survival rate of >90% or >95% not reached within available follow‐up period with reliable estimates for conditional survival.

### 3.3 Gastric neuroendocrine tumors (G‐NETs)

For patients with G‐NETs, the CCS3 of those who were 65‐74 years of age and those who were older than 75 years was 77.7% and 82.7%, respectively, which was significantly lower than that of patients younger than 65 years (approximately 90%; Figure 3A). The initial differences in the conditional survival rate in all age groups largely disappeared after 4 years of survival. Differences in the CCS3 among localized, regional, and distant disease stages declined over time, especially after patients had survived for 7 years; moreover, little excess mortality was observed in patients with all stages of disease (Figure 4A). In addition, little excess mortality was also observed in patients with all tumor grades of disease after patients had survived for 5 years (Figure 5A).

### 3.4 Small intestinal neuroendocrine tumors (SI‐NETs)

Although the mortality rate of elderly patients with SI‐NETs (older than 55 years of age) was still high after 10 years of survival, patients who were younger than 55 years maintained scarcely any excess mortality after diagnosis (Figure 3B). Although the difference in survival rate among the 3 stages (ie, localized, regional and distant) decreased over time, the difference remained until the patients had survived for 10 years (Figure 4B). Note that the difference between grade I, grade II, and grade III/IV disappeared after having survived for 5 years (Figure 5B).

### 3.5 Appendiceal neuroendocrine tumors (A‐NETs)

For patients with A‐NETs who were younger than 45 years, almost no excess mortality was observed since diagnosis, whereas for patients older than 45 years, a similar trend was observed only after 3 years (Figure 3C). The CCS was fairly similar between the localized and regional stage groups, and the survival rate after diagnosis was similar to that of the general population. Although patients with distant disease had a significantly poorer survival at diagnosis than those with localized or regional disease (0‐year CCS3: 98.4% vs 97.2% vs 66.0%), the difference disappeared after 6 years of survival (Figure 4C). Compared with grade I and grade II A‐NETs patients, grade III/IV patients showed most pronounced changes in CCS3 over time (Figure 5C).

### 3.6 Colonic neuroendocrine tumors (C‐NETs)

The CCS3 at diagnosis was significantly lower for patients with C‐NETs who were older than 75 years (54.1%) than for patients younger than 65 years (0‐year CCS3 > 80%; Figure 3D). Although the survival difference between these age groups decreased with the prolongation of survival time, the excess mortality rate in the elderly group was still higher. Patients who were younger than 65 years had almost no excess mortality 2‐3 years after diagnosis, whereas this trend was not observed in patients older than 65 years even after 10 years of survival. The survival rates of those with localized, regional, and distant disease were also significantly different at diagnosis (Figure 4D). The differences between the localized and regional disease groups disappeared after 4 years, whereas survival differences between those 2 groups and the distant disease group remained high. After 5 years of survival, the differences in the conditional survival rate between the localized and regional stage groups disappeared.

### 3.7 Rectal neuroendocrine tumors (R‐NETs)

Among patients with R‐NETs, the CS in all age groups was comparable, and the mortality rate was similar to that of the general population after 2 years of survival (Figure 3E). The difference in survival among the different staging groups persisted (Figure 4E). Although the survival rate of patients with distant disease increased significantly with a prolongation of survival time, the excess mortality remained substantial. Moreover, patients with localized disease exhibited hardly any excess mortality after diagnosis, which indicates a survival similar to that of the general population. Patients with grade III/IV disease exhibited little excess mortality after 4 years.

### 3.8 Pancreatic neuroendocrine tumors (P‐NETs)

A significant difference was found between age groups in terms of the CCS3 at diagnosis for patients with P‐NETs (Figure 3F). Although the survival difference between different age groups decreased with time, excess mortality remained high among patients except for patients older than 75 years who exhibited scarcely any excess mortality after 9 years. Significant differences in survival could also be observed among the 3 different stage groups (Figure 4F). These differences persisted until 10 years after diagnosis, even though they became somewhat smaller over time. Similarly, excess mortality remained substantial for all grade groups.

## 4 DISCUSSION

GEP‐NETs, which are the most frequent subtype of NETs, occur equally in males and females, although the incidence in females has been higher than that in males since 2000 (APC = 5.0 vs 4.0). From 2000 to 2014, the highest incidence of GEP‐NETs has been recorded among Black population in the SEER database (Figure 1G). Regarding the tumor sites, the most frequent sites of GEP‐NETs are the small intestine and the rectum. However, because A‐NETs and P‐NETs show the highest growth trend (APC values of 22.3 and 9.8, respectively), this distribution might change.

Most importantly, although GEP‐NETs are rare (representing approximately 5.84% of all GEP tumors), the incidence of this disease is increasing (APC = 4.5, 95% CI 4.2‐4.8). Notably, according to the SEER data, the rate of this increase is notably higher than the reported incidence of most other neoplasms.^21^, ^22^ The reason for this inverse trend compared with the incidence of general GEP tumors (APC = −2.0, 95% CI −2.0 to −1.9) is unclear. Although it appears that improved histological classification and diagnostic methods might be contributing factors,^23^ the biological characteristics of the tumors might also account for this difference.

Of all the recent studies, this is the first one to focus on the changes in the CCS3 up to 10 years after the GEP‐NET diagnosis. The most up‐to‐date analyses of conditional survival are becoming increasingly necessary due to the marked increase in the number of long‐term GEP‐NET survivors. The current results indicate that CCS estimates for patients with GEP‐NETs were dynamic and increased with time elapsed. Specifically, we are concerned about age‐specific and stage‐specific differences when the CCS approaches 100%, which indicates a lack of excess mortality among cancer patients relative to that in the general population.

Using long‐term follow‐up data from population‐based cancer registries, we were able to analyze the latest and most detailed CCS data for gastroenteropancreatic neuroendocrine tumors at different sites. These results give us a better understanding of the excess mortality rate per year in terms of the survival of patients with GEP‐NETs. We can assume that when the patient’s CCS3 is more than 95%, the survival time is similar to that of individuals of the same age in the general population. Accordingly, we found that NETs occurred in the stomach, rectum, or appendix of all patients, whereas NETs in young individuals were located in the small intestine or colon; NETs in elderly patients would be similar to those in the general population over a certain period of time within 10 years after diagnosis. For the other sites considered, the CCS3 did not exceed 70%‐94%, which indicates that even if these patients survive for a specified time, the survival rate of these patients is still worse than that of the average age group. A higher mortality rate due to a greater comorbidity among patients, late adverse effects of treatment, late recurrences, and secondary tumors might be potential explanations for these observations.

The 3‐year cancer‐specific survival rates of young patients are often better than those of older ones in most tumor types.^12^, ^24^, ^25^, ^26^, ^27^, ^28^ In our study, younger patients with NETs were also found to have a better prognosis than older patients, which is consistent with the observations of other studies.^29^, ^30^ Nevertheless, age‐specific variations in the long‐term CCS3 of patients with GEP‐NETs have not been evaluated in detail. Based on our results, the CS still varied according to age for up to 10 years after diagnosis, even though the effect of age usually decreased with time. However, these results need to be further validated when the stage at diagnosis is considered.

The CSS was also significantly different for the various stage groups at diagnosis. Previous studies have shown that the difference in survival among patients with different stages of stomach, small intestine, appendix, colorectal, and pancreatic cancer decreases with increase in the survival time since diagnosis.^31^, ^32^, ^33^, ^34^, ^35^ However, the tumor stage is still an important prognostic factor, even for conditional survival after 2‐5 years. In studies among patients with various cancers, differences among stage groups decreased or even disappeared over time since diagnosis.^36^ We also observed that the difference among patients with GEP‐NETs in different stage groups decreases with time. In particular, patients with poor prognostic characteristics exhibited a substantial increase in CCS based on actual survival time. Although the data of the SEER histologic grade information in most GEP‐NETs patients are missing in the SEER database, patients who initially had the least favorable tumor grade had the most pronounced CCS3 changes. Thus, the assessment of CCS might be of greater clinical significance for high‐risk patients who have survived for a specified period after diagnosis. In fact, risk predictors identified at the time of diagnosis do not account for the time that has already passed, and patients who survived for a certain period may have a favorable prognosis despite their initial higher risk.

Moreover, both Spanish‐Hispanic‐Latino and Non‐Spanish‐Hispanic‐Latino patients had similar cancer‐specific survival regardless of the location of the tumor, and 2 communities have similar trends of conditional cancer‐specific survival (Figures S6 and S7). Further study about the incidence and survival of the GEP‐NETs in the Spanish‐Hispanic‐Latino and Non‐Spanish‐Hispanic‐Latino population through more detailed large sample data in the future is necessary.

Finally, it must be admitted that our study is not devoid of limitations. We recognized that the SEER database provides a unique opportunity for researchers to test the hitherto unknown medical hypotheses on an unprecedented large amount of patient data and that population‐based epidemiological analysis can be performed at the same time. However, underreporting is a potential limitation of this databank. Moreover, missing data and the evolving definition of GEP‐NETs could have led to misreporting, which might have generated potential selection bias. In addition, the grade of tumor was defined based on the differentiation of the tumor in the SEER database, regardless of the Ki‐67 index, a marker of cellular proliferation, and the mitotic index, which is important for the grading of tumors. Therefore, a large‐scale detailed cohort is necessary to elucidate this problem in the future study. However, our study also has important strengths that must be noted. This is a population‐based study, which might give rise to concerns about the generalizability of the findings, but we believe that the size of the present study is the largest to date and thus that the study can provide comprehensive information on the incidence trend of GEP‐NETs in the USA.

In conclusion, the incidence of GEP‐NETs continues to increase in the USA. In addition, the prognosis of NET survivors generally improves with each year of survival. Patients with NETs in the stomach, rectum, and appendix, as well as younger patients with NETs in the small intestine or colon, hardly experience any excess mortality after a certain time. In fact, CCS estimates for patients with GEP‐NETs improve markedly over time, particularly among patients with initial poor prognoses. Thus, the understanding and application of conditional survival can provide more survival information for patients, clinicians, and researchers, who can then use this information to make life plans and monitor the disease intensity during follow‐up after a GEP‐NET diagnosis.

## CONFLICT OF INTEREST

The authors made no disclosures.

## References

[1] Modlin IM , Oberg K , Chung DC , et al. Gastroenteropancreatic neuroendocrine tumours. Lancet Oncol. 2008;9:61‐72. 18177818 10.1016/S1470-2045(07)70410-2

[2] Modlin IM , Kidd M , Latich I , Zikusoka MN , Shapiro MD . Current status of gastrointestinal carcinoids. Gastroenterology. 2005;128:1717‐1751. 15887161 10.1053/j.gastro.2005.03.038

[3] Frilling A , Åkerström G , Falconi M , et al. Neuroendocrine tumor disease: an evolving landscape. Endocr Relat Cancer. 2012;19:163‐185. 10.1530/ERC-12-0024 22645227

[4] Halperin DM , Shen C , Dasari A , et al. Frequency of carcinoid syndrome at neuroendocrine tumour diagnosis: a population‐based study. Lancet Oncol. 2017;18:525‐534. 28238592 10.1016/S1470-2045(17)30110-9 PMC6066284

[5] PubMed . https://www.ncbi.nlm.nih.gov/pubmed/?term=Neuroendocrine+tumors.

[6] Yao JC , Hassan M , Phan A , et al. One hundred years after “carcinoid”: epidemiology of and prognostic factors for neuroendocrine tumors in 35,825 cases in the United States. J Clin Oncol. 2008;26:3063. 18565894 10.1200/JCO.2007.15.4377

[7] Fraenkel M , Kim M , Faggiano A , de Herder WW , Valk GD . Incidence of gastroenteropancreatic neuroendocrine tumours: a systematic review of the literature. Endocr Relat Cancer. 2014;21:R153. 24322304 10.1530/ERC-13-0125

[8] Dasari A , Shen C , Halperin D , et al. Trends in the incidence, prevalence, and survival outcomes in patients with neuroendocrine tumors in the United States. JAMA Oncol. 2017;3:1335‐1342. 28448665 10.1001/jamaoncol.2017.0589 PMC5824320

[9] Kulke MH , Siu LL , Tepper JE , et al. Future directions in the treatment of neuroendocrine tumors: consensus report of the National Cancer Institute Neuroendocrine tumor clinical trials planning meeting. J Clin Oncol. 2011;29:934. 21263089 10.1200/JCO.2010.33.2056 PMC3068065

[10] Kunz PL , Reidy‐Lagunes D , Anthony LB , et al. Consensus guidelines for the management and treatment of neuroendocrine tumors. Pancreas. 2013;42:557‐577. 23591432 10.1097/MPA.0b013e31828e34a4 PMC4304762

[11] Kulke MH , Shah MH , Benson AB 3rd , et al. Neuroendocrine tumors, version 1.2015. J Natl Compr Canc Netw. 2015;13:78. 25583772 10.6004/jnccn.2015.0011

[12] Janssen‐Heijnen ML , Gondos A , Bray F , et al. Clinical relevance of conditional survival of cancer patients in europe: age‐specific analyses of 13 cancers. J Clin Oncol. 2010;28:2520. 20406936 10.1200/JCO.2009.25.9697

[13] Donovan RJ , Carter OB , Byrne MJ . People’s perceptions of cancer survivability: implications for oncologists. Lancet Oncol. 2006;7:668. 16887484 10.1016/S1470-2045(06)70794-X

[14] Luo G , Javed A , Strosberg JR , et al. Modified staging classification for pancreatic neuroendocrine tumors on the basis of the American Joint Committee on Cancer and European Neuroendocrine Tumor Society Systems. J Clin Oncol. 2017;35:274‐280. 27646952 10.1200/JCO.2016.67.8193

[15] Howlader N , Noone AM , Krapcho M , et al. Surveillance, epidemiology, and end results (SEER) program (http://www.seer.cancer.gov) SEER*Stat Database: incidence ‐ SEER 18 Regs Research Data + Hurricane Katrina Impacted Louisiana Cases, Nov 2014 Sub (2000‐2012) <Katrina/Rita Population Adjustment> ‐ Linke 2014.

[16] Kim Y , Margonis GA , Prescott JD , et al. Curative surgical resection of adrenocortical carcinoma: determining long‐term outcome based on conditional disease‐free probability. Ann Surg. 2017;265:197‐204. 28009746 10.1097/SLA.0000000000001527 PMC4974140

[17] Skuladottir H , Olsen JH . Conditional survival of patients with the four major histologic subgroups of lung cancer in Denmark. J Clin Oncol. 2003;21:3035‐3040. 12915592 10.1200/JCO.2003.04.521

[18] Wang P , Sun Z , Wang W , et al. Conditional survival of patients with gastric cancer who undergo curative resection: a multi‐institutional analysis in China. Cancer. 2018;124:916‐924. 29205321 10.1002/cncr.31160

[19] Beahrs O , Henson DE , Hutter RVP , Myers MH . Manual for Staging of Cancer, 3rd edn Philadelphia, PA: J.B.Lippincott company; 1977.

[20] Pasaoglu E , Dursun N , Ozyalvacli G , Hacihasanoglu E , Behzatoglu K , Calay O . Comparison of World Health Organization 2000/2004 and World Health Organization 2010 classifications for gastrointestinal and pancreatic neuroendocrine tumors. Ann Diagn Pathol. 2015;19:81‐87. 25702616 10.1016/j.anndiagpath.2015.01.001

[21] Torre LA , Bray F , Siegel RL , Ferlay J , Lortettieulent J , Jemal A . GLOBOCAN 2012: estimated cancer incidence mortality and prevalence worldwide in 2012; 2015.

[22] Siegel RL , Miller KD , Jemal A . Cancer statistics, 2016. CA Cancer J Clin. 2016;66:7. 26742998 10.3322/caac.21332

[23] Hallet J , Law CH , Cukier M , Saskin R , Liu N , Singh S . Exploring the rising incidence of neuroendocrine tumors: a population‐based analysis of epidemiology, metastatic presentation, and outcomes. Cancer. 2015;121:589‐597. 25312765 10.1002/cncr.29099

[24] Janssen‐Heijnen ML , Houterman S , Lemmens VE , Louwman MW , Maas HA , Coebergh JW . Prognostic impact of increasing age and co‐morbidity in cancer patients: a population‐based approach. Crit Rev Oncol Hematol. 2005;55:231‐240. 15979890 10.1016/j.critrevonc.2005.04.008

[25] Inghelmann R , Grande E , Francisci S , et al. National estimates of cancer patients survival in Italy: a model‐based method. Tumori. 2005;91:109‐115. 15948535 10.1177/030089160509100201

[26] Bossard N , Velten M , Remontet L , et al. Survival of cancer patients in France: a population‐based study from The Association of the French Cancer Registries (FRANCIM). Eur J Cancer. 2007;43:149‐160. 17084622 10.1016/j.ejca.2006.07.021

[27] Quaglia A , Capocaccia R , Micheli A , Carrani E , Vercelli M . A wide difference in cancer survival between middle aged and elderly patients in Europe. Int J Cancer. 2007;120:2196‐2201. 17285582 10.1002/ijc.22515

[28] Maso LD , Guzzinati S , Buzzoni C , et al. Long‐term survival, prevalence, and cure of cancer: a population‐based estimation for 818 902 Italian patients and 26 cancer types. Ann Oncol. 2014;25:2251‐2260. 25149707 10.1093/annonc/mdu383 PMC4207730

[29] Mosquera C , Koutlas NJ , Fitzgerald TL . Localized high‐grade gastroenteropancreatic neuroendocrine tumors: defining prognostic and therapeutic factors for a disease of increasing clinical significance. Eur J Surg Oncol. 2016;42:1471‐1477. 27528467 10.1016/j.ejso.2016.07.137

[30] Mcmullen T , Al‐Jahdali A , De GC , Ghosh S , Mcewan A , Schiller D . A population‐based study of outcomes in patients with gastrointestinal neuroendocrine tumours. Can J Surg. 2017;60:7616. 10.1503/cjs.007616 PMC5453762 28327275

[31] Wang SJ , Emery R , Fuller CD , Kim JS , Sittig DF , Thomas CR . Conditional survival in gastric cancer: a SEER database analysis. Gastric Cancer. 2007;10:153‐158. 17922092 10.1007/s10120-007-0424-9

[32] Anderson LA , Tavilla A , Brenner H , et al. Survival for oesophageal, stomach and small intestine cancers in Europe 1999‐2007: results from EUROCARE‐5. Eur J Cancer. 2015;51:2144‐2157. 26421818 10.1016/j.ejca.2015.07.026 PMC5729902

[33] Chang GJ , Hu CY , Eng C , Skibber JM , Rodriguezbigas MA . Practical application of a calculator for conditional survival in colon cancer. J Clin Oncol. 2009;27:5938‐5943. 19805670 10.1200/JCO.2009.23.1860 PMC6669907

[34] Wang SJ , Wissel AR , Luh JY , Fuller CD , Kalpathy‐Cramer J , Thomas CR . An interactive tool for individualized estimation of conditional survival in rectal cancer. Ann Surg Oncol. 2011;18:1547‐1552. 21207162 10.1245/s10434-010-1512-3 PMC3156394

[35] Rajeev R , Sekigami Y , Johnston F , Gamblin TC , Turaga KK . Conditional survival as a patient centered metric for patients with appendiceal adenocarcinoma. The Tenth International Symposium on Regional Cancer Therapies; 2015.

[36] Janssen‐Heijnen ML , Houterman S , Lemmens VE , Brenner H , Steyerberg EW , Coebergh JW . Prognosis for long‐term survivors of cancer. Ann Oncol. 2007;18:1408. 17693654 10.1093/annonc/mdm127

