# Supplementary figures and images for: Incidence trend and conditional survival estimates of gastroenteropancreatic neuroendocrine tumors: A large population‐based study
Source: Cancer Med. 2018 Jun 5;7(7):3521–33. doi: 10.1002/cam4.1598 (PMC6051181; doi:10.1002/cam4.1598)

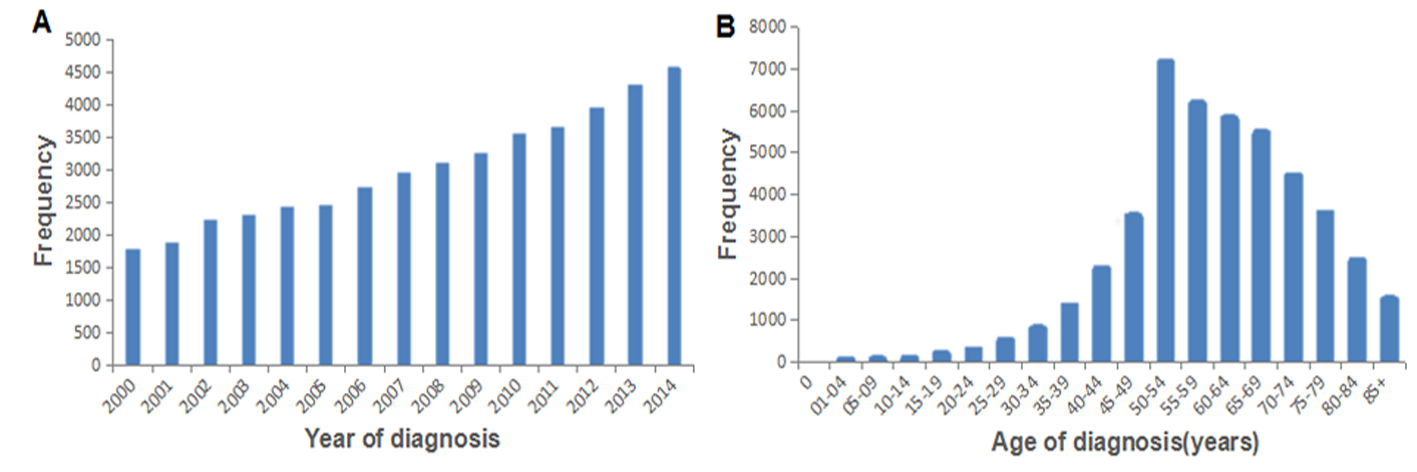

Supplement: Supplementary file 1 [file CAM4-7-3521-s001.tif]

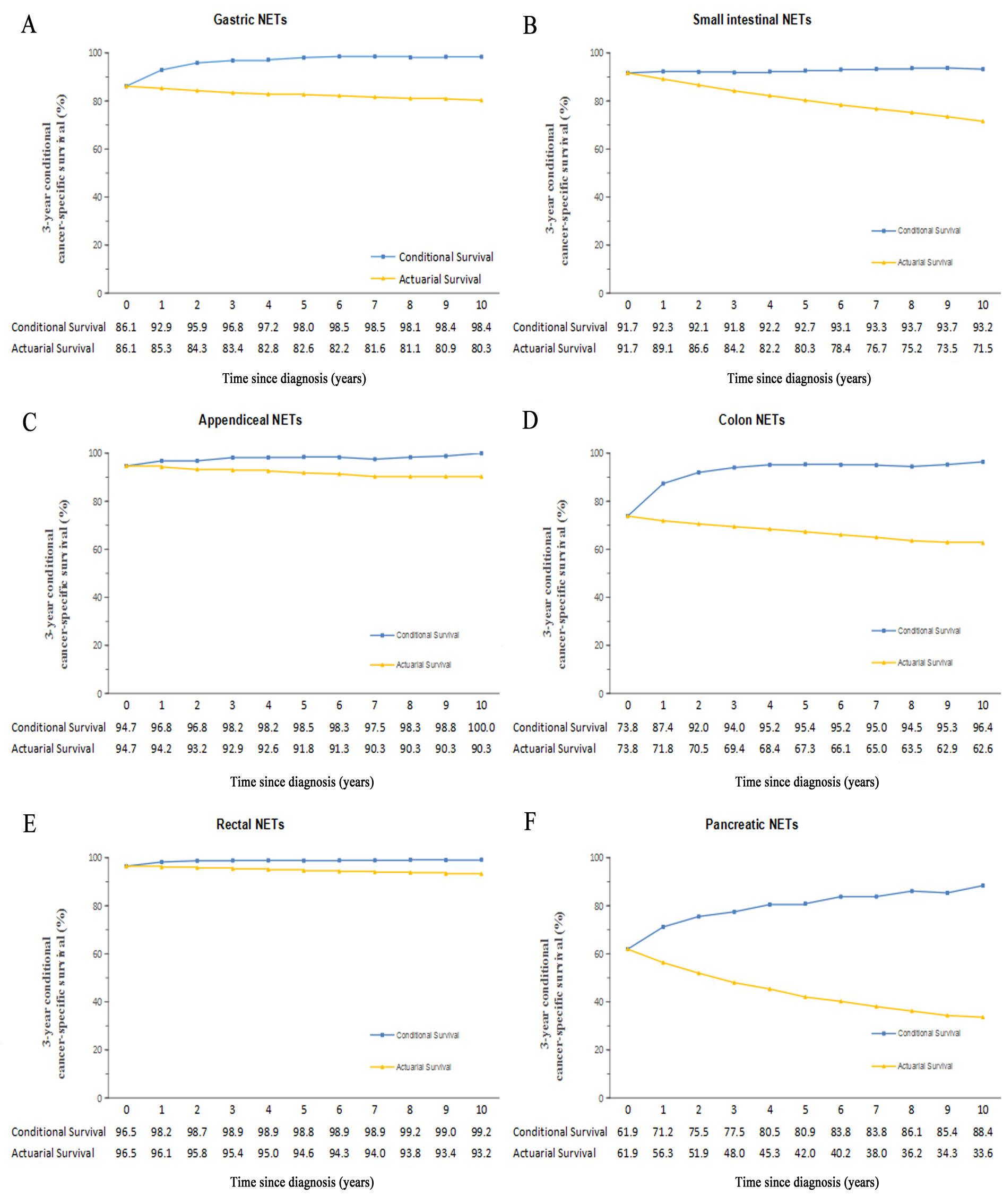

Supplement: Supplementary file 2 [file CAM4-7-3521-s002.tif]

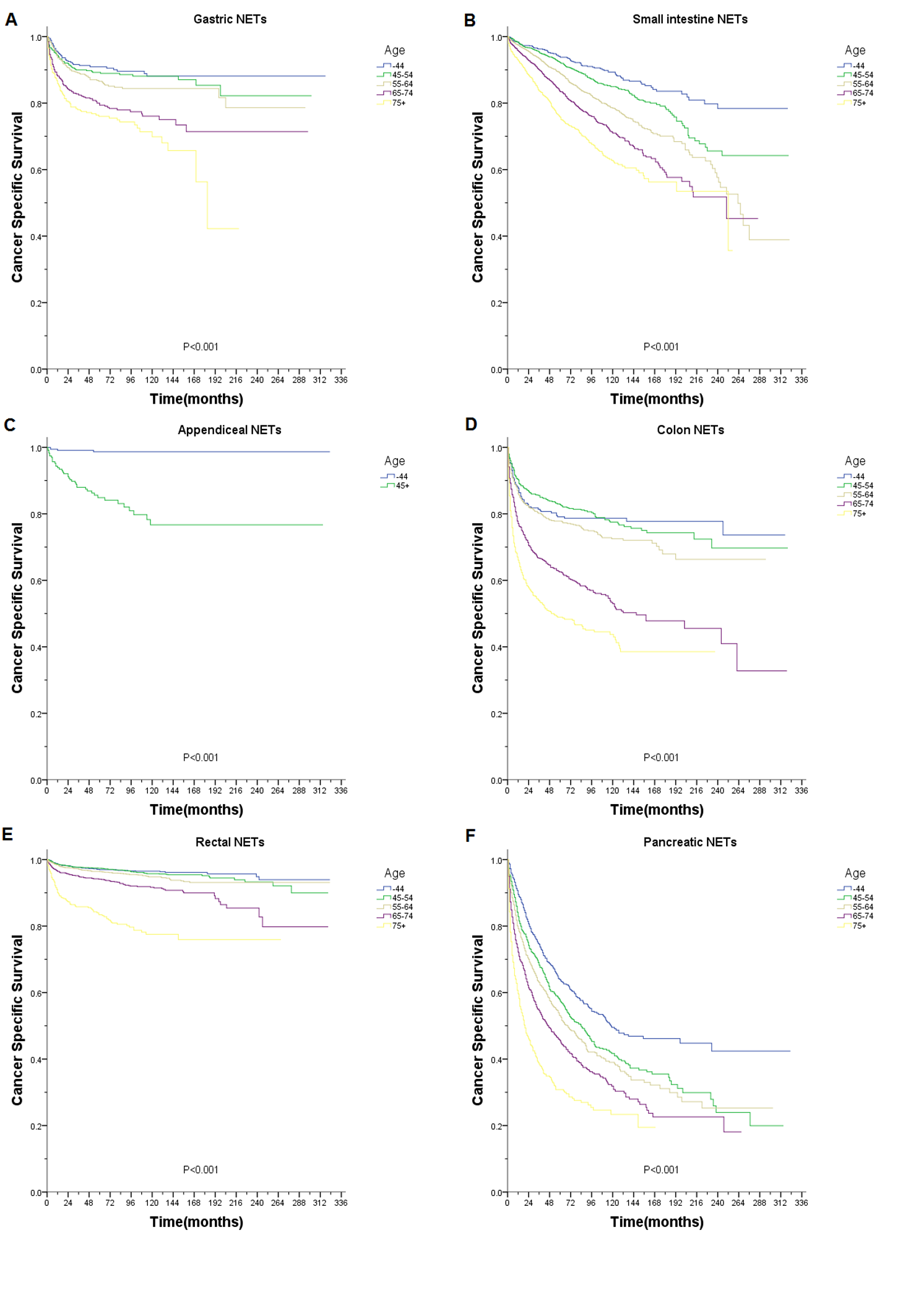

Supplement: Supplementary file 3 [file CAM4-7-3521-s003.tif]

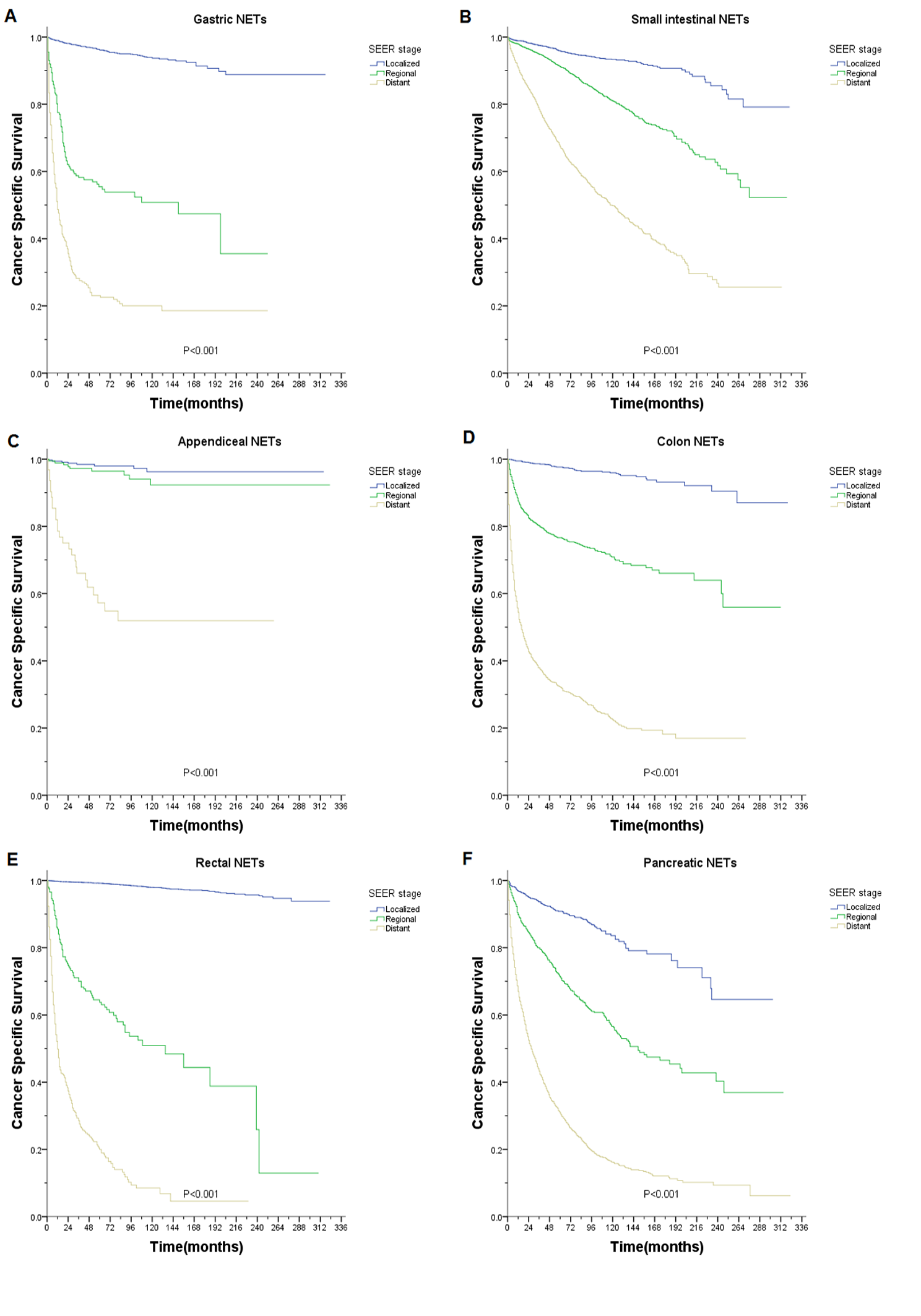

Supplement: Supplementary file 4 [file CAM4-7-3521-s004.tif]

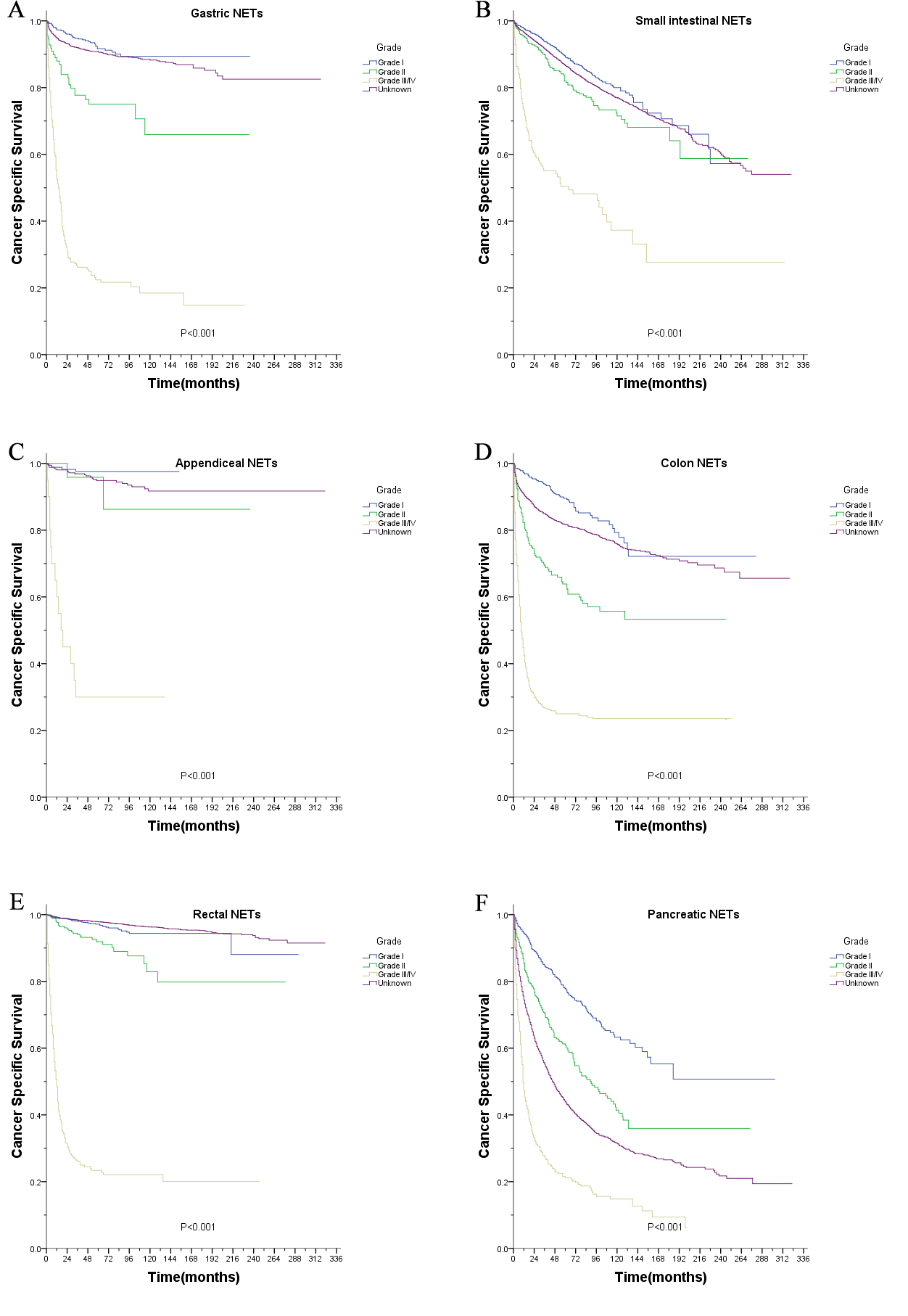

Supplement: Supplementary file 5 [file CAM4-7-3521-s005.tif]

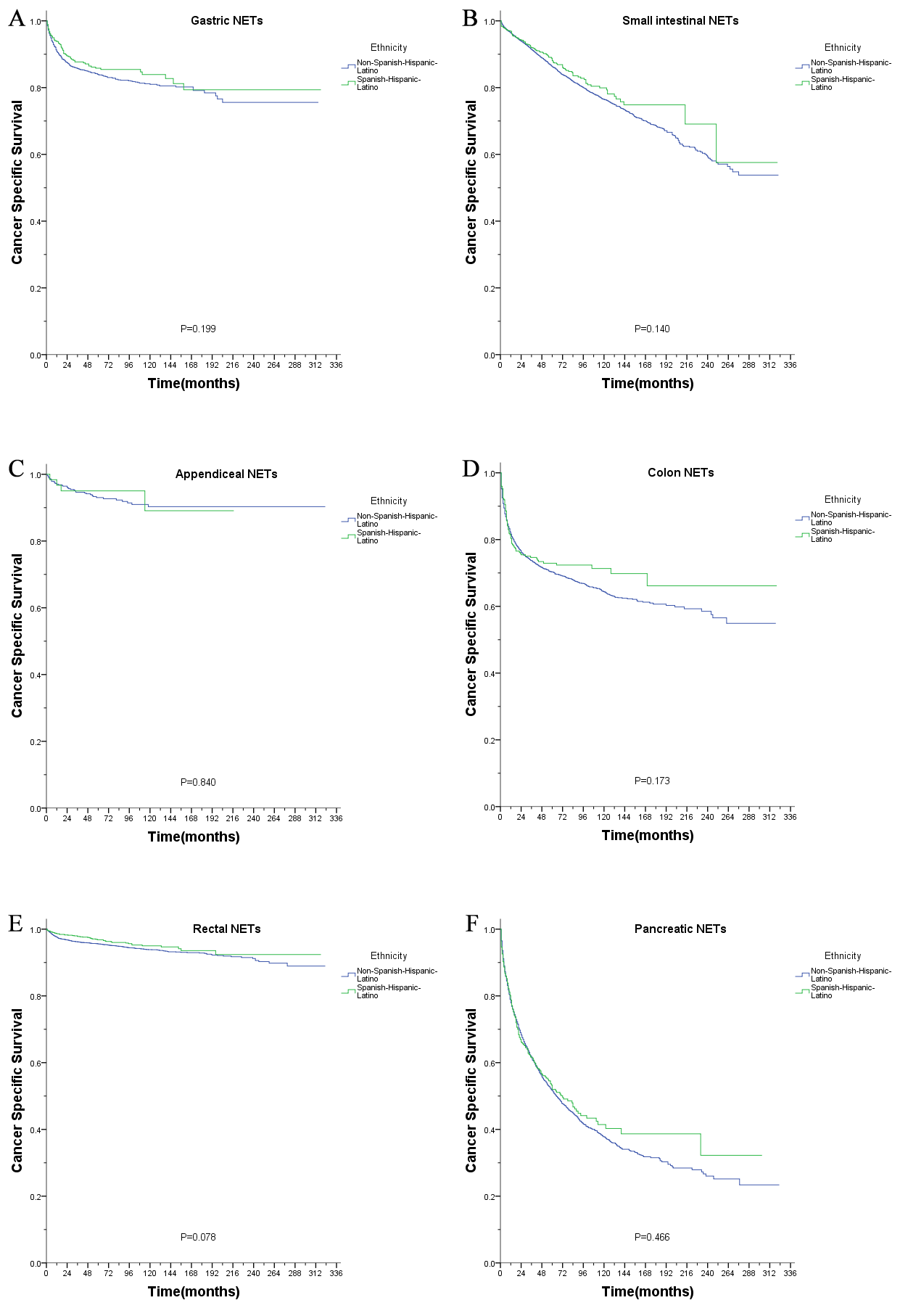

Supplement: Supplementary file 6 [file CAM4-7-3521-s006.tif]

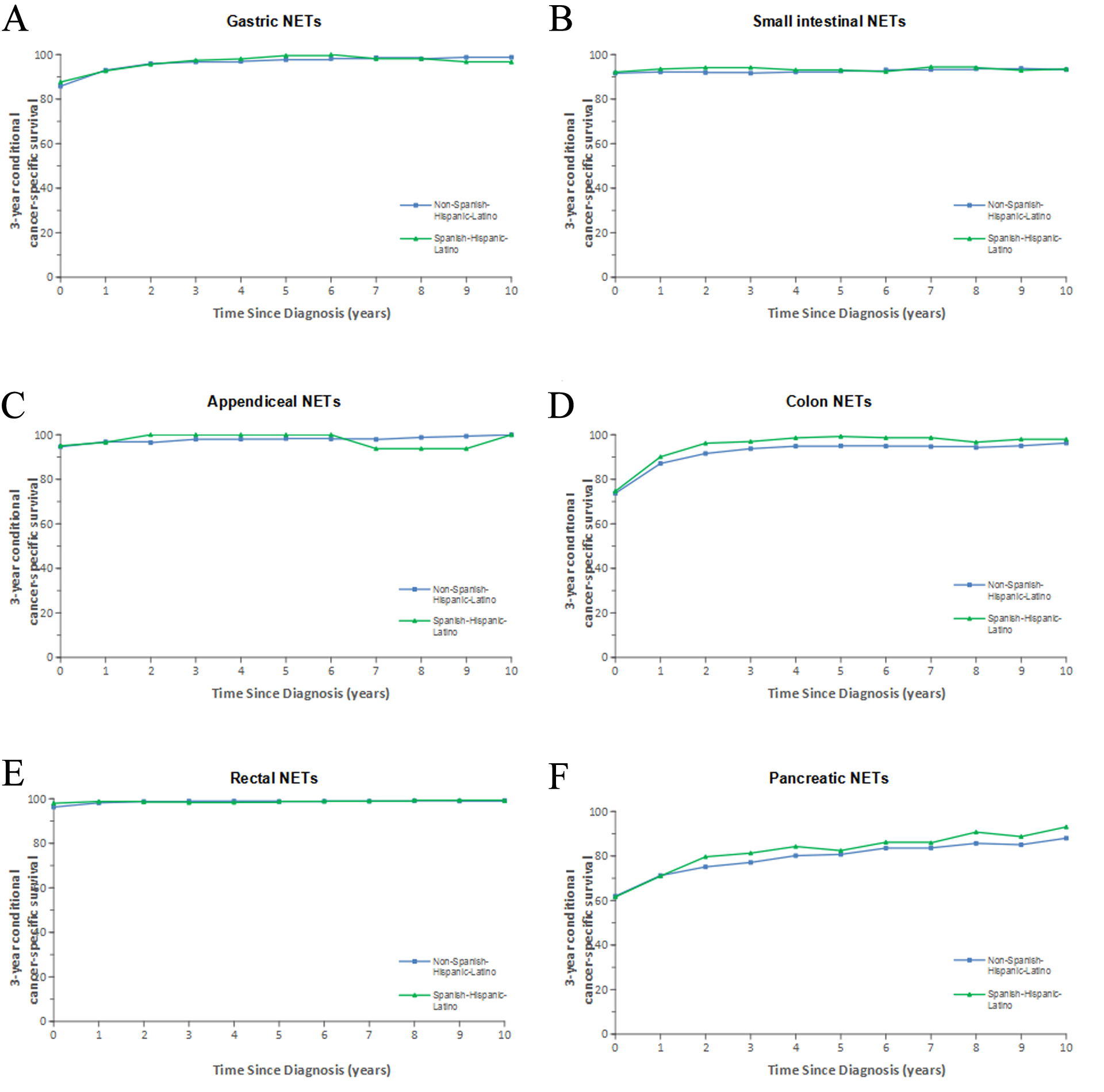

Supplement: Supplementary file 7 [file CAM4-7-3521-s007.tif]
