# Supplementary material for: Incidence trend and conditional survival estimates of gastroenteropancreatic neuroendocrine tumors: A large population‐based study
Source: Cancer Med. 2018 Jun 5;7(7):3521–33. doi: 10.1002/cam4.1598 (PMC6051181; doi:10.1002/cam4.1598)
Supplement: Supplementary file 8 [file CAM4-7-3521-s008.doc]

Supplementary Table 1. Sociodemographic and Clinicopathologic Variables of Gastroenteropancreatic NETs Patients for Conditional Survival Analysis

| Variable | Stomach | | SmallIntestine | | Appendix | | Colon | | Rectum | | Pancreas | |
| --- | --- | --- | --- | --- | --- | --- | --- | --- | --- | --- | --- | --- |
|  | （n=2,608） | | （n=8,637） | | （n=578） | | （n=2,992） | | （n=9,290） | | （n=3,951） | |
|  | No. | % | No. | % | No. | % | No. | % | No. | % | No. | % |
| **Demographic** |  |  |  |  |  |  |  |  |  |  |  |  |
| **CHSDA Region** |  |  |  |  |  |  |  |  |  |  |  |  |
| Alaska | 4 | .2 | 2 | .0 | 1 | .2 | 4 | .1 | 24 | .3 | 5 | .1 |
| East | 948 | 36.3 | 3205 | 37.1 | 334 | 57.8 | 1058 | 35.4 | 2891 | 31.1 | 1328 | 33.6 |
| Northern Plains | 290 | 11.1 | 1277 | 14.8 | 65 | 11.2 | 452 | 15.1 | 1062 | 11.4 | 553 | 14.0 |
| Pacific Coast | 1174 | 45.0 | 3509 | 40.6 | 161 | 27.9 | 1306 | 43.6 | 4790 | 51.6 | 1864 | 47.2 |
| Southwest | 192 | 7.4 | 644 | 7.5 | 17 | 2.9 | 172 | 5.7 | 523 | 5.6 | 201 | 5.1 |
| **Race** |  |  |  |  |  |  |  |  |  |  |  |  |
| White | 2066 | 79.2 | 7022 | 81.3 | 501 | 86.7 | 2357 | 78.8 | 5416 | 58.3 | 3202 | 81.0 |
| Black | 348 | 13.3 | 1322 | 15.3 | 50 | 8.7 | 464 | 15.5 | 2064 | 22.2 | 432 | 10.9 |
| Others | 167 | 6.4 | 248 | 2.9 | 17 | 2.9 | 139 | 4.6 | 1428 | 15.4 | 306 | 7.7 |
| Unknown | 27 | 1.0 | 45 | .5 | 10 | 1.7 | 32 | 1.1 | 382 | 4.1 | 11 | .3 |
| **Ethnicity** |  |  |  |  |  |  |  |  |  |  |  |  |
| Non-Spanish-Hispanic-Latino | 2142 | 82.1 | 8019 | 92.8 | 515 | 89.1 | 2718 | 90.8 | 8121 | 87.4 | 3561 | 90.1 |
| Spanish-Hispanic-Latino | 466 | 17.9 | 618 | 7.2 | 63 | 10.9 | 274 | 9.2 | 1169 | 12.6 | 390 | 9.9 |
| **Sex** |  |  |  |  |  |  |  |  |  |  |  |  |
| Female | 1517 | 58.2 | 4184 | 48.4 | 364 | 63.0 | 1582 | 52.9 | 4628 | 49.8 | 1823 | 46.1 |
| Male | 1091 | 41.8 | 4453 | 51.6 | 214 | 37.0 | 1410 | 47.1 | 4662 | 50.2 | 2128 | 53.9 |
| **Age y** |  |  |  |  |  |  |  |  |  |  |  |  |
| -44 | 374 | 14.3 | 794 | 9.2 | 345 | 59.7 | 289 | 9.7 | 1450 | 15.6 | 693 | 17.5 |
| 45-54 | 516 | 19.8 | 1705 | 19.7 | 99 | 17.1 | 700 | 23.4 | 3226 | 34.7 | 921 | 23.3 |
| 55-64 | 625 | 24.0 | 2336 | 27.0 | 78 | 13.5 | 768 | 25.7 | 2594 | 27.9 | 1021 | 25.8 |
| 65-74 | 598 | 22.9 | 2143 | 24.8 | 42 | 7.3 | 682 | 22.8 | 1476 | 15.9 | 813 | 20.6 |
| 75+ | 495 | 19.0 | 1659 | 19.2 | 14 | 2.4 | 553 | 18.5 | 544 | 5.9 | 503 | 12.7 |
| **Tumor presentation** |  |  |  |  |  |  |  |  |  |  |  |  |
| **SEER stage** |  |  |  |  |  |  |  |  |  |  |  |  |
| Localized | 2095 | 80.3 | 3169 | 36.7 | 333 | 57.6 | 1112 | 37.2 | 8759 | 94.3 | 823 | 20.8 |
| Regional | 200 | 7.7 | 3191 | 36.9 | 182 | 31.5 | 926 | 30.9 | 204 | 2.2 | 823 | 20.8 |
| Distant | 313 | 12.0 | 2277 | 26.4 | 63 | 10.9 | 954 | 31.9 | 327 | 3.5 | 2305 | 58.3 |
| **Grade** |  |  |  |  |  |  |  |  |  |  |  |  |
| Grade I | 528 | 20.2 | 2064 | 23.9 | 170 | 29.4 | 417 | 13.9 | 1190 | 12.8 | 985 | 24.9 |
| Grade II | 111 | 4.3 | 496 | 5.7 | 25 | 4.3 | 210 | 7.0 | 259 | 2.8 | 353 | 8.9 |
| Grade III/Grade IV | 233 | 8.9 | 140 | 1.6 | 20 | 3.5 | 595 | 19.9 | 240 | 2.6 | 355 | 9.0 |
| Unknown | 1736 | 66.6 | 5937 | 68.7 | 363 | 62.8 | 1770 | 59.2 | 7601 | 81.8 | 2258 | 57.2 |

CHSDA, Contract Health Service Delivery Area; SEER, Surveillance, Epidemiology, and End Results;
